# Evidence of SARS-CoV-2 spike protein on retrieved thrombi from COVID-19 patients

**DOI:** 10.1186/s13045-022-01329-w

**Published:** 2022-08-16

**Authors:** Manuela De Michele, Giulia d’Amati, Martina Leopizzi, Marta Iacobucci, Irene Berto, Svetlana Lorenzano, Laura Mazzuti, Ombretta Turriziani, Oscar G. Schiavo, Danilo Toni

**Affiliations:** 1 Emergency Department, Stroke Unit, Sapienza University of Rome, Viale del Policlinico, 155, 00161 Rome, Italy; 2 Department of Radiology, Oncology and Pathological Science, Sapienza University of Rome, Rome, Italy; 3 Department of Medico-Surgical Sciences and Biotechnologies, Sapienza University of Rome, Latin, Italy; 4 Department of Human Neurosciences, Neuroradiology Unit, Sapienza University of Rome, Rome, Italy; 5 Department of Human Neurosciences, Sapienza University of Rome, Rome, Italy; 6 Department of Clinical and Molecular Medicine, Sant’Andrea Hospital, Sapienza University of Rome, Rome, Italy; 7 Department of Molecular Medicine, Sapienza University of Rome, Rome, Italy

## Abstract

The pathophysiology of COVID-19-associated coagulopathy is complex and not fully understood. SARS-CoV-2 spike protein (SP) may activate platelets and interact with fibrin(ogen). We aimed to investigate whether isolated SP can be present in clots retrieved in COVID-19 patients with acute ischemic stroke (by mechanical thrombectomy) and myocardial infarction. In this pilot study, we could detect SP, but not nucleocapsid protein, on platelets of COVID-19 patients’ thrombi. In addition, in all three COVID-19 thrombi analyzed for molecular biology, no SARS-CoV-2 RNA could be detected by real-time polymerase chain reaction. These data could support the hypothesis that free SP, besides the whole virus, may be the trigger of platelet activation and clot formation in COVID-19.

**Supplementary Information:**

The online version contains supplementary material available at 10.1186/s13045-022-01329-w.

## To the Editor,

Thrombotic complications are common features of coronavirus disease 2019 (COVID-19), but the underlying pathogenesis is not fully elucidated yet. It has been observed that the spike protein (SP), namely the protruding membrane protein of SARS-CoV-2, may activate the coagulation cascade by binding angiotensin-converting enzyme 2 (ACE2) directly on platelets and/or endothelial cells [1]. Additionally, the isolated circulating SP may induce a hypercoagulability status by directly interacting with fibrin/fibrinogen [2]. Noteworthy, free SP fragments have been found in plasma of COVID-19 patients [3]. SARS-CoV-2 has been detected rarely in thrombi retrieved from brain arteries of acute ischemic stroke (AIS) patients [4] and more frequently in those retrieved from coronary arteries of acute myocardial infarct (AMI) patients [5]. Few data on SP detection in thrombi retrieved from stroke patients have been reported [6]. We aimed to investigate whether isolated SP can be present in clots retrieved by endovascular treatment in COVID-19 patients with AIS and AMI.

The study was conducted on patients admitted to the Emergency Department of Policlinico Umberto I Hospital, University of Rome La Sapienza, from March 2020 to April 2021. Among a series of consecutive adult patients with large vessel occlusion (LVO)-related AIS or with AMI and a concomitant diagnosis of COVID-19, we retrospectively selected patients with thrombus available for histological analysis. The diagnosis of COVID-19 was based on the positive results of SARS-CoV-2 on real-time reverse transcription polymerase chain reaction (RT-PCR) analysis of nasopharyngeal swab specimens. Unfortunately, we do not have data on the SARS-CoV-2 strain because we did not perform the sequencing for the COVID-19 patients included in the study. However, considering the period (April and October/November 2020) when diagnosis of SARS-CoV-2 infection was made, it is very likely that COVID-19 in these patients was caused by the initial Wuhan variant of the virus. As control, we used thrombi retrieved from patients with LVO AIS not affected by COVID-19. After collection, thrombi were immediately fixed in 10% formalin and embedded in paraffin. Sections were stained with hematoxylin and eosin. Immunohistochemical staining was performed using two different antibodies: SARS-CoV-2 SP (rabbit polyclonal anti-SARS-CoV-2 SP—Cell Signaling Technology, Boston, MA, USA, cat. #56996, dil. 1:100) and nucleocapsid protein (NP) (monoclonal anti-SARS/SARS-CoV-2 (B46F)—Invitrogen, Rockford USA, MA1-7404, dil. 1:200). The positive control consisted of a lung section available from a patient with COVID-19. A double immunofluorescence was performed to colocalize platelets with SARS-CoV-2 SP, using the primary antibodies, anti-CD61 (Monoclonal Mouse Anti-Human CD61, Platelet Glycoprotein IIIa/APC, Clone Y2/51, dil. 1:100) and anti-SARS-CoV-2 SP, visualized, respectively, with Goat anti-Mouse Alexa Fluor 594 (dil. 1:300) and donkey anti-rabbit Alexa Fluor 488 (dil. 1:300) (Thermo Fisher). The nuclei were stained with DAPI. Morphologic and immunohistochemical findings were assessed by two of the authors (GD and ML).

SARS-CoV-2 RNA extraction from clots was carried out by using Total RNA Purification Kit (Norgen Biotek Corp.), according to the manufacturer’s instructions. Viral RNA was amplified for the qualitative detection of SARS-CoV-2 RNA using a real-time RT-PCR system (FTD SARS-CoV-2 test, Siemens Healthineers), as previously described [8].

We enrolled four COVID-19-positive patients: three with LVO AIS (mean age 67 [± 11] years; 3 males) (one out of three patients was also treated with intravenous thrombolysis) and one affected by AMI (43 years old, male). All COVID-19 patients had lung ground-glass opacity on pulmonary CT scan. We included a control group of four LVO AIS without COVID-19 (mean age 69.8 [± 11] years; 3 males), with three of them receiving intravenous thrombolysis (Additional file 1: Table S1).

**Fig. 1:**
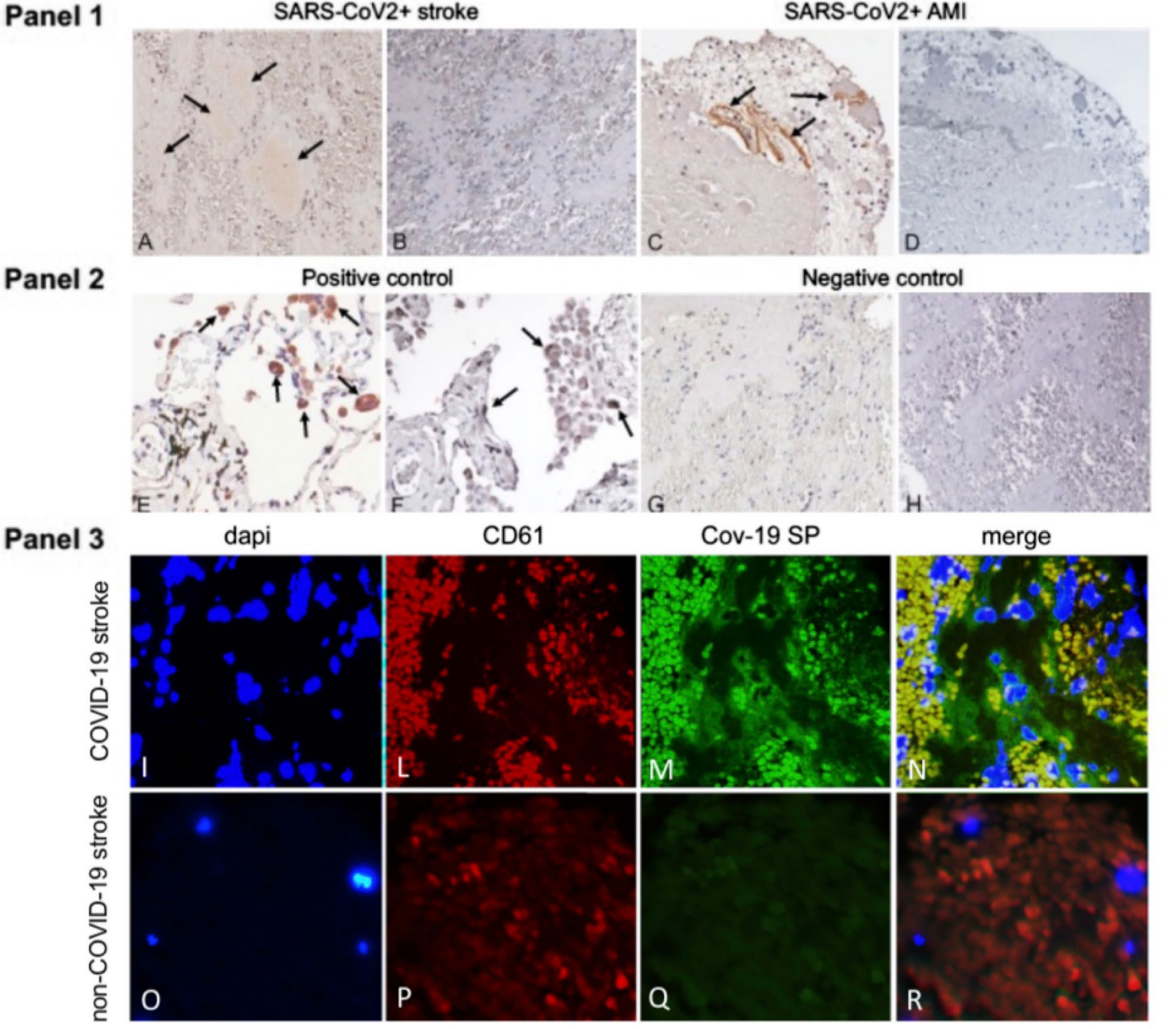
Arterial thrombi from COVID-19 + patients contain SARS-CoV-2 SP but not N protein. Panel 1. Immunostaining positive for SARS-CoV-2 Spike protein (SP) (arrows) in representative thrombotic material from COVID-19 + patients, retrieved from cerebral (A) and coronary (C) arteries. Immunohistochemistry for nucleocapsid protein (NP) was negative in the same samples (B-D). Panel 2. Representative immunohistochemical staining positive for SP (E) and NP (F) (arrows) in the lung of a patient affected by COVID-19 (positive control). Representative immunostaining negative for SP (G) and NP (H) in a thrombus retrieved from the middle cerebral artery of a patient not affected by COVID-19 (negative control). Original magnification 20X. Panel 3. Double immunofluorescence of thrombotic material retrieved from COVID-19 and non-COVID-19 patients’ cerebral arteries. In the COVID-19 thrombus, platelets are co-stained with anti-CD61 (red—L,P) and anti-SARS-CoV-2 spike protein (SP) antibodies (green—M,Q), emitting yellow signals in the merged panel (N), while in the control (non-COVID thrombus) only the red CD61 signal is observed (R)

The relative amount of platelets and fibrin/red blood cells did not significantly differ from COVID-19 thrombi and controls. COVID-19 thrombi retrieved from cerebral arteries showed mild positivity for SP, whereas SP immunostaining was more marked in the COVID-19 thrombus retrieved from anterior descending coronary artery (Fig. 1, Panel 1 A, C). Interestingly, in the clot of the AMI patient, immunostaining for CD61 was patchy, yet substantially overlapped with SP (Additional file 1: Fig. S1). Neither cerebral nor coronary artery thrombi showed cells positive for NP (Fig. 1, Panel 1 B, D). As for comparison, Fig. 1 Panel 2 reports representative immunohistochemical staining for SP and NP which was positive for both (E and F, respectively) in the lung of a patient affected by COVID-19 (positive control) and negative (G and H, respectively) in a thrombus retrieved from the middle cerebral artery of a patient not affected by COVID-19 (negative control).

Finally, to characterize the cellular population expressing SP we performed a double-immunostaining with antibody against CD61 and we found that most of the SP colocalized with platelets (Fig. 1, Panel 3).

No SARS-CoV-2 RNA could be identified in three COVID + thrombi analyzed with RT-PCR.

No specific differences in the coagulation parameters (Table 1) as well as in demographics and clinical characteristics (Additional file 1: Table S1) were observed between AIS with and without COVID-19 and between AIS patients and the AMI patient with COVID-19. Except for the common vascular risk factors, based on our available data, apparently, there were no other possible specific triggers of thrombosis. In particular, none of the study patients was on heparin therapy prior to the admission (Table 1).

**Table 1:** Coagulation parameters of the study patients

	LVO AIS with COVID-19 n = 3	LVO AIS without COVID-19 n = 4	AMI N = 1	Normal range
PLT count, × 10^3^/μL			633	150–450
(n. of pts with available data)	(3/3)	(4/4)		
Mean	199.33	231.0		
Median	184.0	228.0		
MPV, fL			8.0	7.2–13.0
(n. of pts with available data)	(3/3)	(4/4)		
Mean	9.5	8.1		
Median	9.5	8.2		
INR			1.31	0.8–1.2
(n. of pts with available data)	(3/3)	(2/4)		
Mean	1.01	1.0		
Median	0.95	1.0		
aPTT			0.83	0.8–1.2
(n. of pts with available data)	(3/3)	(2/4)		
Mean	0.89	0.94		
Median	0.96	0.92		
d-dimer, μg/L	n/a		390	0–550
(n. of pts with available data)		(3/4)		
Mean		1646.33		
Median		805.0		
Fibrinogen, μg/dL			277	200–400
(n. of pts with available data)	(3/3)	(3/4)		
Mean	476.33	345.33		
Median	556.00	334.0		
ATIII, %	n/a	n/a	94	80–120
(n. of pts with available data)				
Mean				
Median				
FVIII, %			n/a	58–130
(n. of pts with available data)	(1/3)	(3/4)		
Mean	83.30	55.97		
Median		54.90		
vWFAg, %			n/a	41–130
(n. of pts with available data)	(1/3)	(3/4)		
Mean	221.90	136.33		
Median		138.0		
vWFRCo, %			n/a	41–124
(n. of pts with available data)	(1/3)	(3/4)		
Mean	261.60	127.67		
Median		140.90		
FXIII, %			n/a	64–140
(n. of pts with available data)	(1/3)	(3/4)		
Mean	107.80	83.10		
Median		82.50		

Proportions in round brackets represent the number of patients with available data

aPTT, activated partial thromboplastin time; INR, international normalized ratio; MPV, mean platelet volume; n/a, not available; PLT, platelet; vWFAg, von Willebrand factor antigen; vWFRCo, von Willebrand factor ristocetin cofactor

The main limitation of this study was the very limited sample size which prevented us from drawing definite conclusions. However, in our opinion, the present data could support the hypothesis that free SP, besides the whole virus, may be the trigger of platelet [1, 2] and endothelial [6] activation, and clot formation in COVID-19. This event could precede or run in parallel with the recently hypothesized spike-specific immune-complex (IC)-mediated platelet activation in COVID-19 critically ill patients [9, 10]. An aberrant glycosylation of these ICs also seems to increase platelet thrombus formation [11]. Another limitation of our study is that we did not perform confocal microscopy analysis. To the best of our knowledge, only one other group has recently looked at the presence of SP in thrombi retrieved from 6 AIS patients, nevertheless, with negative results [7]. The different type of anti-SP antibodies used (monoclonal versus polyclonal in our study) as well as the different burden of COVID-19 on stroke pathogenesis may be plausible explanations. In addition, a possibly diverse genetically determined ACE2 receptor expression on platelets and endothelial cells could also justify the different chance to find SP in clots. Larger studies are needed to confirm our findings.

## Supplementary Information

**Additional file 1**. *Supplementary Table.* Demographics and other clinical characteristics of the study patients. *Supplementary Figure.* Representative images of serial sections of the clot from the COVID-19 patient with acute myocardial infarction, stained for spike protein (SP) and CD61 for platelets identification.

## Data Availability

The data of this report are available from the corresponding author upon reasonable requests.

## Abbreviations

SP: Spike protein
RT-PCR: Reverse transcription polymerase chain reaction
ACE2: Angiotensin-converting enzyme 2
COVID-19: Coronavirus disease 2019
AIS: Acute ischemic stroke
AMI: Acute myocardial infarct
LVO: Large vessel occlusion
NP: Nucleocapsid protein
