# Supplementary material for: Evidence of SARS-CoV-2 spike protein on retrieved thrombi from COVID-19 patients
Source: J Hematol Oncol. 2022 Aug 16;15:108. doi: 10.1186/s13045-022-01329-w (PMC9380658; doi:10.1186/s13045-022-01329-w)
Supplement: Supplementary file 1 — Additional file 1. Supplementary Table. Demographics and other clinical characteristics of the study patients. Supplementary Figure. Representative images of serial sections of the clot from the COVID-19 patient with acute myocardial infarction, stained for spike protein (SP) and CD61 for platelets identification. [file 13045_2022_1329_MOESM1_ESM.pdf]

ADDITIONAL FILE

Evidence of SARS-CoV-2 Spike protein on retrieved thrombi from COVID-19 patients

Manuela De Michele (MD, phD, MSc)[1], Giulia d'Amati (MD, phD)[2], Martina Leopizzi (MD, phD)[3], Marta Iacobucci (MD)[4], Irene Berto (MD)[1], Svetlana Lorenzano (MD, phD, MSc)[5], Laura Mazzuti (MD, phD)[6], Ombretta Turriziani (MD, phD) [7], Oscar G. Schiavo (MD)[1], Danilo Toni (MD, phD)[5]

[1]Emergency Department, Stroke Unit, Sapienza University of Rome, Rome, Italy

[2] Department of Radiology, Oncology and Pathological Science, Sapienza University of Rome, Rome, Italy

[3]Department of Medico-surgical sciences and Biotechnologies, Sapienza University of Rome, Latin, Italy

[4] Neuroradiology Unit, Department of Human Neurosciences, Sapienza University of Rome, Rome, Italy

[5] Department of Human Neurosciences, Sapienza University of Rome, Rome, Italy

[6] Department of Clinical and Molecular Medicine, Sapienza University, Sant'Andrea Hospital

[7] Department of Molecular Medicine, Sapienza University

Running heads: SARS-CoV-2 Spike protein in COVID-19 clots

Corresponding Author:
Manuela De Michele
Sapienza University of Rome - Emergency Department, Stroke Unit
Viale del Policlinico, 155 - 00161
Rome, Italy
Phone numbers: +39/0649979595; +39/0649979529
Email: m.demichele@policlinicoumberto1.it

Supplementary Table. Demographics and other clinical characteristics of the study patients

	LVO AIS patients with COVID-19<br><br>n=3	LVO AIS patients without COVID-19<br>n=4	AMI patient<br><br><br>n=1
Demographics			
Age, yrs (n. of pts with available data)<br>- mean	(3/3)<br>67	(4/4)<br>69.8	43
Sex, male	2/3	3/4	1
Vascular risk factors and comorbidities			
Smoking	0	0	-
Hypertension	1/3	1/4	-
Diabetes mellitus	1/3	0	-
Atrial fibrillation	2/3	1/4	-
Dyslipidemia	1/3	1/4	-
Ischemic cardiopathy	0	0	-
Previous stroke/TIA	1/3	0	-
Anemia	1/3	0	-
Hypothyroidism	0	0	-
Carotid artery stenosis >50%	0	2/4	-
Valvulopathy	0	0	-
Neoplasm	0	0	-
Medications prior to admission			
Antiplatelets	1/3	2/4	-
Anticoagulants	0	0	-
Beta-blockers	1/3	0	-
ACE-I/ACEIIR-I	1/3	2/4	-
Diuretics	0	1/4	-
Calcium antagonists	0	0	-
Lipid lowering medications	0	0	-
Blood glucose lowering medications	0	0	-
Admission			
Time from diagnosis of COVID-19 to AIS or AMI onset (days)<br>- mean	(2/3)<br><br>0	Not applicable	24
NIHSS at admission<br>- mean<br>- median	(3/3)<br>11.7<br>8.0	(4/4)<br>19.3<br>21.5	Not applicable
NIHSS at 24 h<br>- mean<br>- median	(3/3)<br>8.0<br>8.0	(4/4)<br>13.5<br>12.5	Not applicable
Artery occlusion<br>- MCA-M1<br>- MCA-M2<br>- ICA-MCA, tandem occlusion<br>- Coronary artery	3/3<br>2<br>1<br>0<br>0	4/4<br>3<br>0<br>1<br>0	 0<br>0<br>0<br>1
IV rt-PA	1/3	3/4	-
Endovascular treatment	3/3	3/4	1
Stroke volume, cm^3<br>- mean<br>- median	3/3<br>20.93<br>8.30	4/4<br>20.05<br>18.00	Not applicable
Pneumonia	2/3	0	1

Abbreviations: ACE-I/ACEIIR-I, Angiotensin Converting Enzyme Inhibitors/Angiotensin Converting Enzyme-II Receptor Inhibitors; AIS, acute ischemic stroke; AMI, acute myocardial infarction; ICA, internal carotid artery; IV, intravenous; MCA, middle cerebral artery; NIHSS, National Institutes of Health Stroke Scale; rt-PA, recombinant tissue plasminogen activator; TIA, transient ischemic attack.

Supplementary Figure.

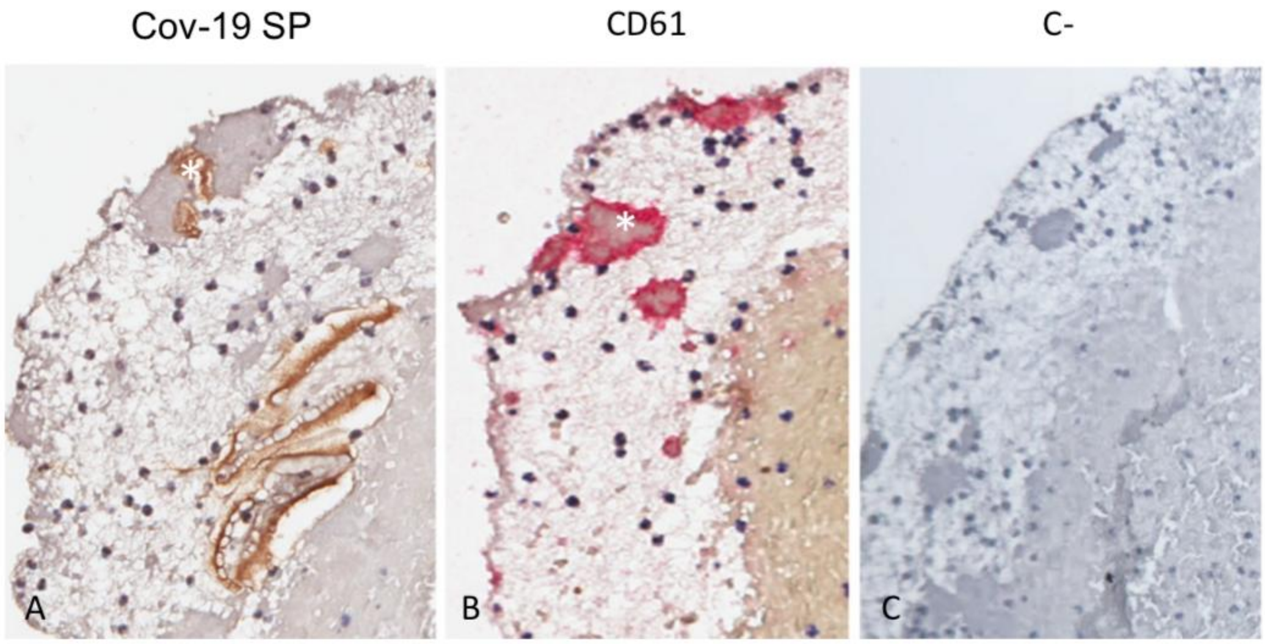

Representative images of serial sections of the clot from the COVID-19 patient with acute myocardial infarction, stained for SP (in brown - A) and CD61 for platelets identification (in red - B). There is a substantial overlap between the patchy areas of CD61 staining and SP p[illegible]ein (white asterisks). C- represents the negative control (omitting the primary antibody).

Abbreviation: Cov-19 SP: SARS-CoV-2 spike protein
